# Conservation in the face of diversity: multistrain analysis of an intracellular bacterium

**DOI:** 10.1186/1471-2164-10-16

**Published:** 2009-01-11

**Authors:** Michael J Dark, David R Herndon, Lowell S Kappmeyer, Mikel P Gonzales, Elizabeth Nordeen, Guy H Palmer, Donald P Knowles, Kelly A Brayton

**Affiliations:** 1 Program in Genomics, Department of Veterinary Microbiology and Pathology, School for Global Animal Health, Washington State University, Pullman, WA 99164-7040, USA; 2 Animal Disease Research Unit, U.S. Department of Agriculture/Agriculture Research Service, Pullman, WA 99164-7030, USA; 3 College of Veterinary Medicine, Western University of Health Sciences, Pomona, CA 91766-1854, USA

## Abstract

**Background:**

With the recent completion of numerous sequenced bacterial genomes, notable advances have been made in understanding the level of conservation between various species. However, relatively little is known about the genomic diversity among strains. We determined the complete genome sequence of the Florida strain of *Anaplasma marginale*, and near complete (>96%) sequences for an additional three strains, for comparative analysis with the previously fully sequenced St. Maries strain genome.

**Results:**

These comparisons revealed that *A. marginale *has a closed-core genome with few highly plastic regions, which include the *msp2 *and *msp3 *genes, as well as the *aaap *locus. Comparison of the Florida and St. Maries genome sequences found that SNPs comprise 0.8% of the longer Florida genome, with 33.5% of the total SNPs between all five strains present in at least two strains and 3.0% of SNPs present in all strains except Florida. Comparison of genomes from three strains of *Mycobacterium tuberculosis, Bacillus anthracis*, and *Nessieria meningiditis*, as well as four *Chlamydophila pneumoniae *strains found that 98.8%–100% of SNPs are unique to each strain, suggesting *A. marginale*, with 76.0%, has an intermediate level of strain-specific SNPs. Comparison of genomes from other organisms revealed variation in diversity that did not segregate with the environmental niche the bacterium occupies, ranging from 0.00% to 8.00% of the larger pairwise-compared genome.

**Conclusion:**

Analysis of multiple *A. marginale *strains suggests intracellular bacteria have more variable SNP retention rates than previously reported, and may have closed-core genomes in response to the host organism environment and/or reductive evolution.

## Background

While the recent boom in genome sequencing projects has provided a wealth of information about bacterial metabolism and evolution, we know little about interstrain variation. A firm understanding of the rates and sites of variation is useful in determining genotypic differences associated with phenotypic traits and in formulating control strategies for a number of pathogens. Further, knowledge about the pan-genome of organisms will aid in determining the core genomic requirements, as well as shed more light on events that occur in the various environmental niches bacteria occupy.

Most studies of bacterial diversity to date have either utilized specific genomic loci [1,2] or have examined metagenomics of specific environmental niches [3,4]. While these types of studies help elucidate the extent of diversity, there is still a key component that has not yet been investigated – a measurement of diversity within bacterial species. Obtaining a true measure of species diversity is difficult, as the strains selected for whole-genome sequencing are generally chosen to examine a particular phenotypic trait, subjecting any resultant measures of diversity to selection bias.

The level of interstrain diversity can have a significant impact on the direction of research. Selection of pathogen strains for sequencing is typically based on differences in virulence [5], host preference [6], or tissue tropism [7]. Using these selection criteria may artificially skew the level of diversity in the studied genome sequences, resulting in a biased level of diversity which does not accurately reflect the true genetic diversity of the species. However, since the diversity among strains has only been examined in a small number of species, determining if there is a skew is difficult. For example, analysis of several genome sequences of *Bacillus anthracis *found a low number of single-nucleotide polymorphisms (SNPs) [8], which led to development of other techniques for examining the epidemiology of outbreak strains [9]. *B. anthracis *is an example of a "closed core" genome – that is, after sequencing several strains (four for *B. anthracis*), no strain-specific genes are added to the pan-genome [10], which may be a result of a clonal population split of *B. anthracis *from *B. cereus*. Thus, the closed-core genome may be the result of small evolutionary distance, and may be a rare finding for organisms with larger evolutionary distance. The alternative is an "open core" genome, where each new sequenced strain adds at least one unique gene to the pan-genome. This is exemplified by *Streptococcus agalactiae*, which has approximately 30 new strain-specific genes for each additional genome sequenced, regardless of the total number of strains compared.

What influences the pan-genome? Is the pan-genome content fixed, or does it drift with time? Do all non-clonal populations have open-core genomes, or is this influenced by the environment a bacterium occupies? While answering all of these questions will require sequencing many more genomes, *Anaplasma marginale *makes an excellent system for studying the last question for a number of reasons. *A. marginale *is a member of the order *Rickettsiales *and a well-established obligate intracellular bacterial model. *A. marginale *is the most globally prevalent vector-borne pathogen of cattle, causing cyclic anemia, decreased production, and possibly death [11]. A previous genome sequence for the St. Maries strain [11] establishes that this organism has a small genome size due to reductive evolution, and is related to several other intracellular pathogens, including those in the genera *Anaplasma*, *Ehrlichia*, and *Rickettsia *[12]. In addition, *A. marginale *has a number of characterized strains, with each strain defined by *msp1α *genotype [13,14]. While previous studies have utilized specific genes to examine differences between these strains [15-17], no studies have examined the species diversity of *A. marginale*. A number of studies have described strains that vary in geographic location and phenotypic traits [15,18,19], and these are available for determination of the true level of genetic diversity in this species, subsequent analysis of the status of core genes, and determination if these are correlated with the intracellular lifestyle, geographic location, tick-transmissible status, or other characteristics of these organisms.

To answer this question, we obtained genome sequences for four strains of *A. marginale *that have differing abilities to be transmitted by *Dermacentor andersoni*, with each phenotype represented by at least two geographically distinct isolations. We sequenced the Florida strain to completion using a BAC-based clone by clone approach, and obtained high coverage genome sequence data for three additional strains. The resulting DNA sequences were then analyzed and compared to both the previously sequenced St. Maries genome [11], as well as other bacterial species in the Order *Rickettsiales*. Further, the genomes of several other non-rickettsial bacteria were examined with similar genome comparison techniques to determine if diversity and pan-genome content are related to pathogenicity or an intracellular lifestyle.

## Results

### Microbial genome diversity

**Figure 1:**
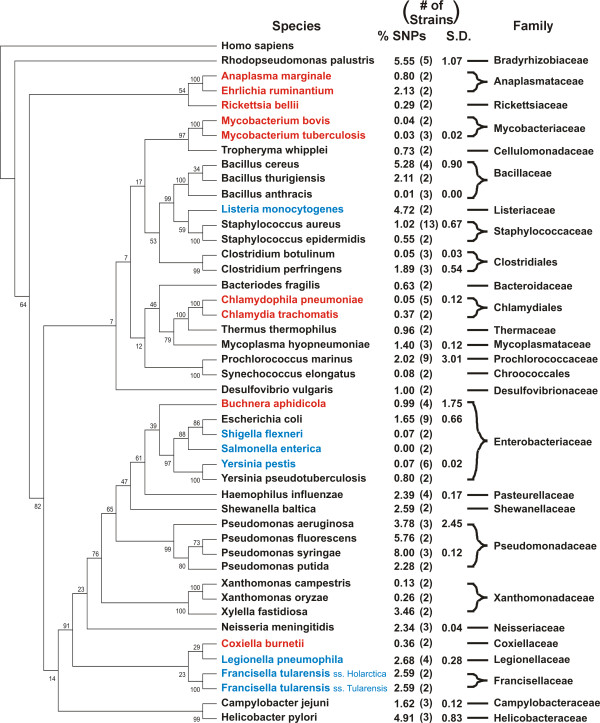
**Comparison of the level of SNP diversity among sequenced genomes**. Bacteria listed in blue are obligate intracellular, while those in red are facultatively intracellular. Each organism lists the average level of SNPs as a percentage of the largest genome.

Previous studies [6,20] have shown high levels of variation between the genomes of different strains of obligate intracellular bacteria. To compare the levels found in *A. marginale *to other genomes, similar comparisons were made for organisms meeting the following criteria: organisms with 1) a single chromosome, 2) more than one sequenced strain, and 3) assembled and finished genome sequences deposited in Genbank, including free-living, facultative intracellular, and obligate intracellular bacteria (Figure 1). Single-factor analysis of variance (ANOVA) finds no significant differences in the level of variation between obligate intracellular, facultative, and free-living bacteria. The number of SNPs ranged from 0.00% to 8.00% of the larger genome, with significant intraspecies and intragenera variation.

### General genome features and comparison of the St. Maries and Florida strains

The *Anaplasma marginale *Florida strain genome is composed of a single 1,202,435 bp circular chromosome predicted to contain 942 coding sequences (CDS) (Table 1). Similar to most other previously sequenced *Anaplasmataceae*, there are no plasmids and no identifiable insertion sequences. Compared to the previously sequenced St. Maries strain genome [11], there are seven fewer CDSs despite the larger genome size, due primarily to differences in split open reading frames (ORFs) and annotation differences. The high degree of synteny between these two strains is disrupted by two inversions; one approximately 30 kb long is flanked by repeat elements (*msp3 *pseudogenes), while the other is a single gene flanked by short duplicated hypothetical genes.

**Table 1:** Comparison of the St. Maries and Florida genome features

	*Anaplasma marginale*	
	St. Maries	Florida
Genome Size (bp)	1,197,687	1,202,435

CDS features	949	942
GC content (%)	49.86%	49.86%
Coding density (%)	85.40%	85.50%
Average gene length	1078	1091

rRNA genes	3	3
tRNA genes	37	37
Functional pseudogenes	16	17

Split ORFs were first described in the *Rickettsia conori *genome [21], and are postulated to represent genes that are in the first stage of reductive evolution. The idea that these ORFs have split recently is consistent with the findings in *Anaplasma*, as different ORFs are split in the two completely sequenced strains. The four split ORFs annotated in the St. Maries genome (*mutL*, *murC*, *aatA*, and *aspS*) [11] are intact in the Florida genome, and two tandem genes annotated as hypothetical in the St. Maries genome (AM574 and AM576) are fused in the Florida genome (AMF_437). Only one split ORF, *petA*, is found in the Florida genome. Four small ORFs in the St. Maries genome (AM380, AM395, AM974, and AM976), ranging in size from 204 bp to 378 bp are not present in the Florida genome. These ORFs are flanked by repetitive DNA sequences, and appear to be missing due to recombination events.

### Genes mediating genome plasticity

**Figure 2:**
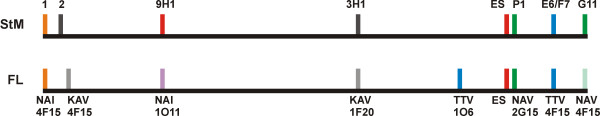
**Physical map of the MSP2 functional pseudogenes in the St. Maries (StM) and Florida (FL) strains**. Vertical bars indicate relative position of functional pseudogenes in the genome (not to scale). Bars with the same color indicate identical functional pseudogenes, while similar colors indicate functional pseudogenes with segmental changes. ES represents the *msp2 *expression site.

The *msp2 *superfamily is a group of related *A. marginale *genes encoding surface proteins [11]. *Msp2 *encodes a highly antigenic protein that varies over time during infection by gene conversion of functional pseudogenes into a single expression site, to create new antigenic variants capable of evading the existing immune response. Compared to the St. Maries genome, the Florida genome has one additional *msp2 *functional pseudogene. Of the eight Florida *msp2 *functional pseudogenes, four are identical to those in the St. Maries genome. The Florida genome has two sets of duplicated functional pseudogenes, TTV 4F15/TTV 1O6 and KAV 4F15/KAV 1F20 (Figure 2); while St. Maries was found to have duplicated functional pseudogenes, this was not noted in a functional pseudogene-targeted examination of other strains [22]. Florida has a set of duplicated functional pseudogenes in the same genome positions as St. Maries (2/3H1 in St. Maries, and KAV 4F15/KAV 1F20 in Florida). As obligate intracellular bacteria are not thought to undergo lateral gene transfer, identical functional pseudogenes indicates the sequence is either evolutionarily conserved or has been selected independently in both strains due to a fitness advantage. Interestingly, both copies in Florida have a change encoding 15 amino acids at the 5' end of the hypervariable region compared to their St. Maries counterparts; either both strains duplicated a functional pseudogene after this change occurred in an ancestral strain, or both copies in one of the strains acquired identical changes after the ancestral strain duplicated the original functional pseudogene. In contrast, only two of the seven MSP3 functional pseudogenes are identical between Florida and St. Maries (*msp3 *C/*msp3*-1, and *msp3 *4L1/*msp3 *6). The *omp1-15 *genes are present in both genomes, with a high degree of conservation between the predicted amino acid sequences (85.3–100% identity) as previously reported [17].

### *Aaap *gene family

**Figure 3:**
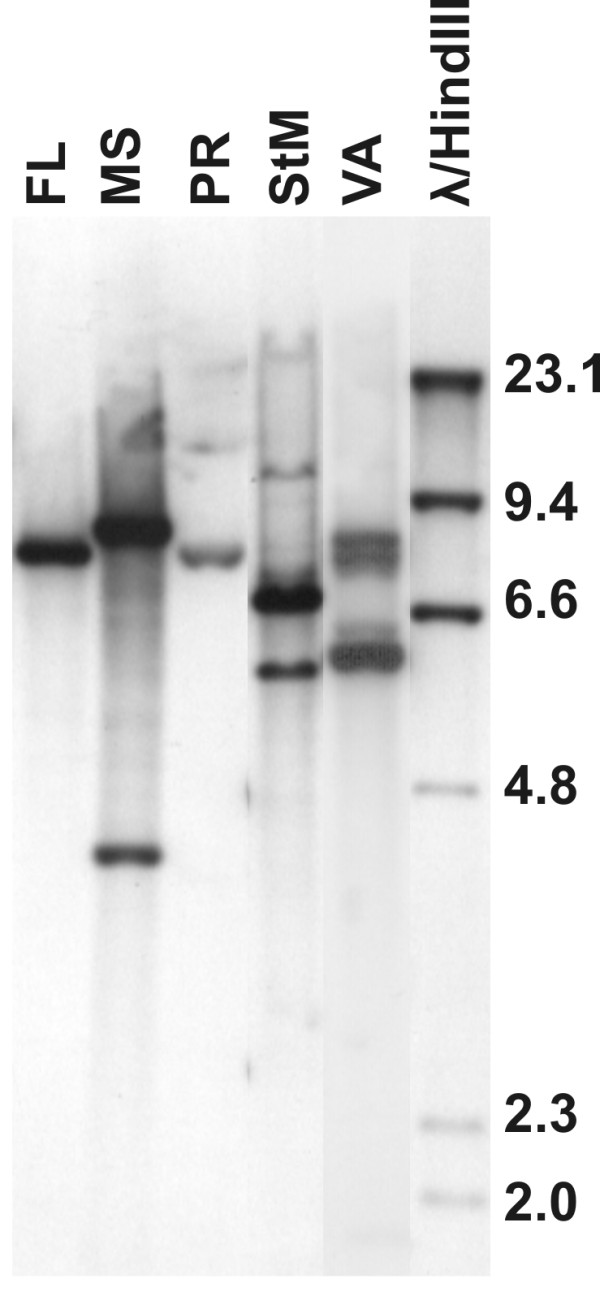
**Southern blot of the aaap locus in *A. marginale *strains**. FL – Florida strain, MS – Mississippi strain, PR – Puerto Rico strain, StM – St. Maries strain, VA – Virginia strain. DNA marker sizes are listed in kbp.

The *aaap *gene was first recognized and characterized as an *Anaplasma *appendage associated protein [23]. Subsequently, additional related genes were identified that appear to be tandemly-duplicated copies that have diverged to have relatively low levels of sequence identity (Table 2). There is expansion of this locus in the Florida strain relative to the St. Maries strain, with a duplicated copy of the *aaap *gene. Because of the repetitive nature of this gene family, these sequences tend to be missing from pyrosequenced genome assemblies; therefore, we examined the status of this locus in several the strains via Southern analysis, revealing that this locus is highly plastic both within and between strains (Figure 3).

**Table 2:** Identity between deduced AAAP amino acid sequences from the St. Maries and Florida strains

		St. Maries		
		*aaap*	AM879	AM880
Florida	*aaap*	45.0%	49.4%	49.6%
	*alp1*	46.1%	73.8%	24.1%
	*alp2*	33.4%	40.3%	58.2%
	*alp3*	11.7%	30.7%	24.6%

### High density sequence coverage of additional strains

**Figure 4:**
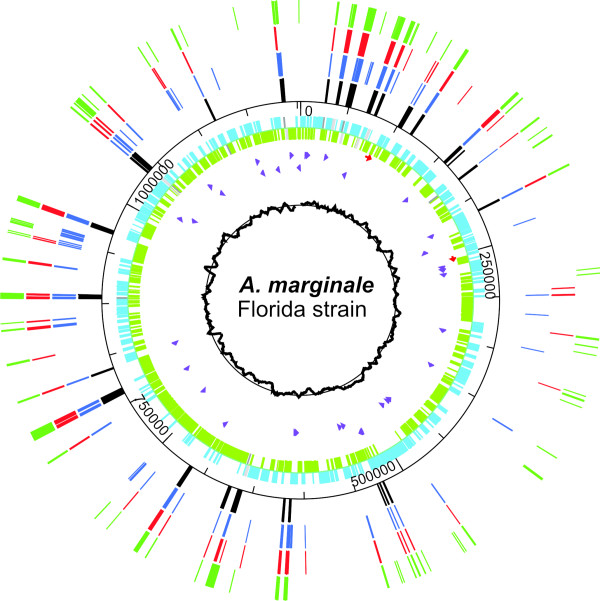
**Distribution of gaps in the three pyrosequenced genomes**. In the outer rings, gaps in the Mississippi sequence are green, gaps in Puerto Rico are red, and gaps in Virginia are blue. Known repetitive genes are represented by black bars. The inner rings represent CDSs (blue and green) and functional pseudogenes (shades of grey) in the Florida strain, rRNAs (red) and tRNAs (purple), and the G-C skew (black graph).

An additional two transmissible strains (Virginia and Puerto Rico) and one non-transmissible strain (Mississippi) were subjected to genome-scale pyrosequencing [24] (454 Life Sciences, Branford, CT), which provided at least 96% genome coverage when compared to either Florida or St. Maries (Table 3). Most of the missing sequences corresponded to repetitive regions (such as *msp2 *and *msp3 *pseudogenes and the *aaap *locus) (Figure 4) [also see additional files 1 and 2], and reflects the limitations of assembling short sequence reads (averaging approximately 250 bp per read) without additional scaffolding. No new genes were detected in the pyrosequenced contigs of any of the strains.

**Table 3:** Pyrosequencing results for three strains of *Anaplasma marginale*

	**Puerto Rico**		**Virginia**		**Mississippi**	
	St. Maries	Florida	St. Maries	Florida	St. Maries	Florida

Number of large contigs	75	59	81	70	78	82
Bases in large contigs	1,150,801	1,158,530	1,146,893	1,153,875	1,139,486	1,141,520
% Genome coverage	96.89%	97.00%	96.88%	96.77%	96.34%	96.25%
High quality variations	6,038	2,729	6,613	3,868	6,302	6,773

### Diversity of *A. marginale *strains

**Figure 5:**
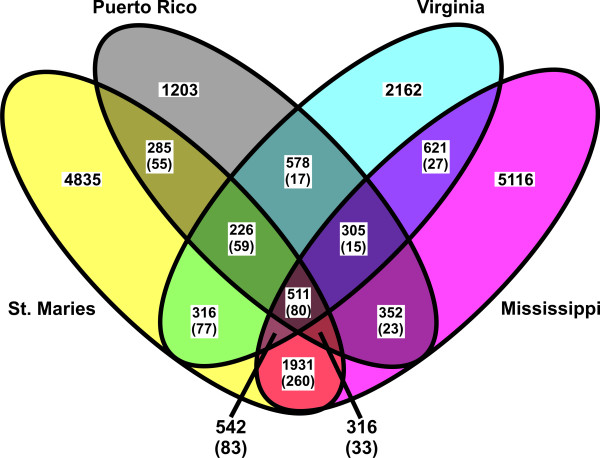
**Distribution of SNPs in four strains compared to the Florida strain**. Numbers in parentheses show SNPs that are different from Florida in each strain in the subset, but are not the same in the compared strains.

Global comparison of all strains with the Florida strain revealed 20,028 total sites with a single nucleotide polymorphism (SNP) in at least one of the compared strains. Of these, 511 (2.6%) were different in the Florida genome and identical in the other four strains, and 13,316 (66.5%) were unique to one of the four strains (Figure 5). The remaining 30.9% of SNPs represent those SNPs relative to Florida that were present in two or three of the strains. There were 9,609 SNPs between the Florida and St. Maries strains, comprising 0.80% of the larger Florida genome. The SNPs were distributed evenly throughout the genome, which is similar to both *Ehrlichia ruminantium *and *Rickettsia bellii *[see additional file 3], and are proportionally distributed throughout coding and non-coding regions. The numbers of polymorphisms in the Puerto Rico, Virginia, and Mississippi strains (2,729, 3,868, and 6,773, respectively) are minimums, as the gaps are regions predicted to have significant numbers of SNPs. When the genome size was corrected for the gaps in coverage, the SNP rates for the Puerto Rico, Virginia, and Mississippi genomes were 0.32%, 0.46%, and 0.73% of the Florida genome, respectively.

## Discussion

This study illustrates the dangers of drawing universal conclusions when strains are selected based on specific criteria, such as phenotypic differences. No two strains in this study are truly representative of the population as a whole. Additionally, the large number of differences in pair-wise comparisons of any two strains illustrates the difficulty of associating genes with phenotypic differences, and the utility of sequencing multiple strains to increase the power of these associations. While our initial selection of the Florida strain was based on a phenotypic difference – that of tick transmissibility, the selection of subsequent strains (PR, VA, and MS) were made to try and minimize the effect of bias based on that phenotype, as well as select a wider geographic range of isolates to increase interstrain diversity. Interestingly, when the pyrosequenced strains are compared to Florida, there are more high-quality polymorphisms (identified when four reads, each with at least 20 base pairs flanking the polymorphic site, contain the difference, with at least one read in each direction) between Florida and the Mississippi strain, despite the fact that neither is tick-transmissible by *D. andersoni *[19,25]. Further, St. Maries appears to be an outlier sequence, as there are at least 6,000 differences between St. Maries and all other sequenced strains.

The level of SNP diversity in these strains coupled with the high degree of gene content conservation also sheds an interesting light on the concept of the "core genome", described for *Streptococcus agalactiae *[10]. For *S. agalactiae*, approximately 90.5% of genes were considered part of the "core genome", or constant between strains, and each new strain added additional strain-specific genes to the "pan-genome". This is contrasted with *Bacillus anthracis*, which had no new strain-specific genes after four strains were compared. The strains of *A. marginale *sequenced here present an interesting data point, as *A. marginale *has not been hypothesized to be a clonal population derived from another organism (as has been postulated for *B. anthracis*), and yet has a closed core genome. The accumulation of large numbers of SNPs might indicate a greater evolutionary distance; however, the closed-core genome could be due to other factors. These could include the isolated nature of the intracellular niche occupied by *A. marginale*, causing the organism to undergo reductive evolution to the point it is approaching the minimal gene complement, or may be, despite our efforts, related to the strains selected for sequencing. However, if this is due to long-term reductive evolution, it calls into question the source of the six split ORFs between the Florida and St. Maries genomes, as these are thought to be early reductive changes. Another possibility is that transmission of the organism among animals in a relatively restricted geographic area (i.e., within a herd) promotes a relatively clonal population of organisms through isolation in a similar environment.

Analysis of the level of SNP diversity in several bacterial genomes brings into question previous conclusions about the variability of obligate intracellular pathogens. Previous studies [6] have found relatively large numbers of SNPs between intracellular organisms. It was therefore hypothesized that the relatively isolated intracellular niche limits opportunities for genetic exchange and increased numbers of SNPs provides a compensatory mechanism for providing diversity to drive evolution. Our results suggest this is unlikely, as there is no correlation between intracellular, facultative intracellular, and free-living organisms and the level of diversity. With few exceptions, there is a large range in the degree of variability in all the strains compared. Additionally, the organisms with the two highest rates of variability, *Pseudomonas syringae *and *Rhodopseudomonas palustris*, are both free-living. There is also significant variation at the genus and family level. These data suggest that the factors for retention of SNPs leading to bacterial diversity are likely multifactorial and complex.

While the composition of the gene content of the pan-genome is obviously important, this study reveals another characteristic that needs examination: the level of diversity in the pan-genome. The minimum of 20,028 variable sites found among these five genomes is approximately 1.67% of the estimated size of the pan-genome. The large number of unique SNPs in each strain (24.1% in the St. Maries genome, 6.0% in the Puerto Rico genome, 10.8% in the Virginia genome, and 25.5% in the Mississippi genome) suggests that while *A. marginale *has a closed core genome, the SNP profile of the core genome is moderately "open". When several strains of *Streptococcus agalactiae *(CJB111, COH1, A909, and 515) are compared to the 2603 VR strain, 99.18% of the 46,579 total detected SNPs are unique to an individual strain, while zero SNPs are common to all four strains. Similarly, 100% of SNPs between three strains of *Bacillus anthracis *(Ames, Ames Ancestor, and Sterne) and *Mycobacterium tuberculosis *(F11, H37Ra, and H37Rv), 98.8% of SNPs between three strains of *Neisseria meningitides *(FAM18, MC58, and Z2491), and 99.9% of SNPs between four strains of *Chlamydophila pneumoniae *(AR39, CWL029, J138, and TW-183) are unique to one strain. This suggests that these genomes have open SNP profiles regardless of being open or closed-core at the genome level. Further, there is no correlation between SNP diversity and lifestyle, with high levels of variation between strains and within genera, with limited exceptions. However, given that the majority of strains were selected based on phenotypic traits or previous work with each strain, it is unlikely that this represents the true diversity of these organisms. Additionally, the majority of organisms have only two sequenced strains, making analysis of variation within a species impossible to determine. Additional work will be required to build a picture of genomic diversity.

The genome of *A. marginale *is highly recombinogenic, which, in spite of the highly conserved gene content, leads to increased plasticity. There are between five and nine functional *msp2 *pseudogenes in the strains examined to date [11,22,26,27], and these can recombine in whole or in part into the *msp2 *expression site (or with each other) to generate new antigenic variants [26,27]. Symmetrical inversions around the origin are thought to be quite common in bacteria [28] and have been noted in *Anaplasmataceae*, often utilizing repeated genes such as *msp2 *to mediate the inversion. These inversions are highlighted by comparisons between *A. marginale *and *Ehrlichia ruminantium *[29] and *Anaplasma phagocytophilum *[30]. Many of these repetitive sequences flank *ori*, as does another duplicated gene, *rho*. While not around the origin, a smaller scale inversion was found between two strains of *A. marginale *flanked by *msp3 *pseudogenes close to *ori*. Another highly plastic genomic region is the AAAP locus [23] that appears to be expanding and contracting within and between strains. In addition to changes in gene number, the sequences are highly variable (Table 2). Further research will be needed to determine the significance of these differences, as well as the function of this locus.

## Conclusion

Sequencing of multiple strains of bacteria, as well as sequencing multiple isolates from the same strain, will yield a tremendous amount of information about natural rates of variation in bacterial populations, which in turn will influence our views of bacterial evolution, epidemiology, and vaccine strategies. This study reveals that interstrain SNP diversity does not appear to be influenced by the environmental niche an organism occupies, nor is it generally consistent throughout a specific family or genera. Comparison of multiple strains of *A. marginale *finds few changes at the gene level, while there is robust diversity at the nucleotide level. Finally, multistrain SNP analysis appears to be a more powerful tool for *A. marginale *phylogenetic studies than genotyping of the major surface proteins [15], and this strategy should be useful for epidemiologic studies of other species of bacteria.

## Methods

### Experimental approval

All animal experiments described in this paper were approved by the Washington State University Institutional Animal Care and Use Committee (IACUC), with approval number 3386.

### Strains of *A. marginale *used

The Florida strain [GenBank: CP001079] of *A. marginale *was originally isolated from a pool of blood samples collected from cattle in 1955 [16,31,32]. The Mississippi strain [GenBank: ABOP00000000] was isolated from an acute clinical case of anaplasmosis [25,33]. Both of these strains are virulent, and are not transmissible by *D. andersoni *ticks. The Virginia strain [GenBank: ABOR00000000] was isolated from a cow in Southern Virginia in 1972 [34]. The Puerto Rico strain [GenBank: ABOQ00000000] was received as a frozen stabilate after isolation from cattle in Puerto Rico in 1985 [35,36]. Both the Virginia and Puerto Rico strains are virulent, and are transmissible by *D. andersoni*. While passage histories are not well documented for these strains, all strains have been passaged multiple times in cattle since isolation. The Florida strain has the longest passage history, being passed continuously since isolation, and the Puerto Rico strain has only been passaged once since coming to our laboratory.

### DNA isolation for genome sequencing

Blood stabilates from the Florida, Mississippi, Puerto Rico, and Virginia strains were inoculated into splenectomized calves, which were shown to be free of *A. marginale *infection via competitive enzyme-linked immunoabsorbent assay (cELISA) [37]. Blood samples were taken at peak parasitemia, washed seven times in phosphate buffered saline (137 mM NaCl, 10 mM Phosphate, and 2.7 mM KCl), and centrifuged at 1,500 × g for 10 minutes with the removal of the buffy coat after each spin. Erythrocytes not used immediately were diluted 1:1 in PBS and frozen for later use.

### *A. marginale *preparation

After thawing, lysed erythrocytes were passed over a column containing loosely-packed CF-11 cellulose (Sigma-Aldrich Corporation, St. Louis, MO). The column eluate was washed repeatedly with PBS and centrifuged at 19,000 g for 20 minutes until all remaining hemoglobin was removed, leaving a pellet of *A. marginale *initial bodies and erythrocyte membranes.

### Bacterial artificial chromosome (BAC) library construction and manipulation

The bacterial preparation was embedded into 1% agarose blocks (A-9539, Sigma Chemical Co., St. Louis, MO), and cells within the blocks were lysed using proteinase K and SDS [38]. *A. marginale *genomic DNA was partially digested with either *Hind*III or *Mbo*I, size selected on pulse-field gels, ligated into the pBELOBAC11 vector, and electroporated into *Escherichia coli *strain DH10B (Amplicon Express, Pullman, WA). A total of 3,072 clones (1,536 clones from each restriction enzyme) were arrayed into 384 well plates. The average insert size of the clones was 120 kb.

### Genome sequencing

For the Florida strain, a BAC-based clone-by-clone strategy was adopted. BAC clones were screened using digoxigenin (DIG)-labeled (Roche Applied Science) probes to bovine genomic DNA and several *A. marginale *genes (including *msp1α*, *msp1β*, *msp2*, *msp3*, *msp4*, *msp5*, *dnaK*, *recA*, *groEL*, and *sodB*). Selected clones were end-sequenced and a minimum tiling path was constructed based on comparison with the previously-sequenced St. Maries strain. Sequencing of BACs, assembly of completed sequences, and genome annotation were as described [11].

Genomic DNA from the Mississippi, Puerto Rico, and Virginia strains was extracted from isolated bacteria (prepared as described above) using the Puregene Blood kit (Qiagen Corporation, Valencia, CA). DNA was then sequenced on a Genome Sequencer 20 instrument (454 Life Sciences Corporation, Branford, CT), using a pyrosequencing protocol [24]. The Newbler program was used with its default settings to assemble the sequence and to compare all contigs to the completed Florida and St. Maries genomes, which revealed the location of gaps in coverage. High-quality variations were called when four reads, each with at least 20 base pairs flanking the polymorphic site, contain the difference, with at least one read in each direction. The nucleotide sequences of assembled contigs were compared to the St. Maries genome using BLASTn. Any contigs without hits better than 1e^-10 ^were then compared to the bovine whole genome shotgun sequence database, to screen for bovine DNA contamination. Any contigs with no hits to bovine sequence were then compared to the nt database. Large contigs assembled by Newbler were compared to the Florida genome using MUMmer v3.1 [39] after filling gaps in the assembly with the corresponding sequence from the Florida strain.

### Genome comparisons

MUMmer v3.1 was used to compare the completed St. Maries and Florida genomes and the contigs of the Mississippi, Puerto Rico, and Virginia strains, as described [5]. Output from the SNP detection algorithm (show-snps) was processed using custom scripts (written with AutoIt v3.2.2.0) to determine the number of SNPs per ORF. Show-snps output was also processed in Excel (Microsoft Corporation, Redmond, WA) to graph the location of SNPs throughout the genome. FASTA sequences from single-chromosome genomes with multiple strains sequenced were downloaded from Genbank [see supplementary table 2 for the genomes compared, their sizes, and Genbank accession numbers]. All strains for a given species were compared to each other using MUMmer 3.1, as described above. The number of SNPs per comparison was then divided by the larger of the two compared genomes to yield the percent SNPs per genome. For species with more than two strains sequenced, all percentages were averaged to give the mean and standard deviation. The phylogenetic tree was inferred using the Maximum Parsimony method [40] of MEGA4 [41] comparing concatenated sequences from *groEL*, *groES*, *atpA*, and *recA*. The bootstrap consensus tree is inferred from 1000 replicates, and branches corresponding to partitions reproduced in less than 50% bootstrap replicates are collapsed. There were a total of 458 positions in the final dataset, out of which 379 were parsimony informative.

### Southern analysis

Genomic DNA from all five strains was digested with *XbaI *and *HindIII *(New England Biolabs Corporation, Ipswich, MA), as these enzymes cut within the conserved flanking genes. Resultant fragments were separated on a 0.8% agarose gel, and subsequently transferred to a charged nylon membrane and crosslinked with a Stratalinker UV apparatus (Stratagene Inc., La Jolla, CA) per the manufacturer's directions. The blots were prehybridized at 42°C for at least two hours in Dig Easy Hyb buffer (Roche Corporation, Indianapolis, IN). Digoxigenin-labeled probes to *aaap *were produced using the PCR DIG Probe Synthesis Kit (Roche Corporation) and hybridized overnight at 42°C in DIG Easy Hyb buffer. The membrane was washed three times for 15 minutes in 2 × SSC and 0.1% SDS, with the first two washes at room temperature and the third at 65°C. A final wash was performed in 0.2 × SSC and 0.1% SDS at 65°C. Chemiluminescent detection of the probes was performed using the DIG Wash and Block Buffer Kit (Roche Corporation) per the manufacturer's directions.

## Authors' contributions

MJD, GHP, DPK, and KAB designed the study. DRH, LSK, KAB, and MJD participated in sequencing and assembly of the Florida genome. MPG, EN, and MJD carried out additional targeted sequencing. MJD isolated DNA for pyrosequencing, and performed computational analysis. MJD, KAB, LSK, DRH, EN, and MPG analyzed data. MJD, GHP, DPK, and KAB wrote and edited the manuscript. All authors read and approved the final manuscript.

## Supplementary Material

Additional file 1 **Pyrosequencing gaps**. A listing of genes containing gaps in the pyrosequenced genomes (Puerto Rico [PR], Virginia [VA], and Mississippi [MS]). Click here for file

Additional file 2 **Species compared in SNP analysis**. A listing of bacterial species compared in the SNP analysis, with Genbank accession numbers for each genome analyzed. Click here for file

Additional file 3 **SNP distribution in three species**. Distribution of SNPs between the compared strains of *A. marginale*, *E. ruminantium*, and *R. bellii*. Click here for file
