# Supplementary figures and images for: Conservation in the face of diversity: multistrain analysis of an intracellular bacterium
Source: BMC Genomics. 2009 Jan 11;10:16. doi: 10.1186/1471-2164-10-16 (PMC2649000; doi:10.1186/1471-2164-10-16)

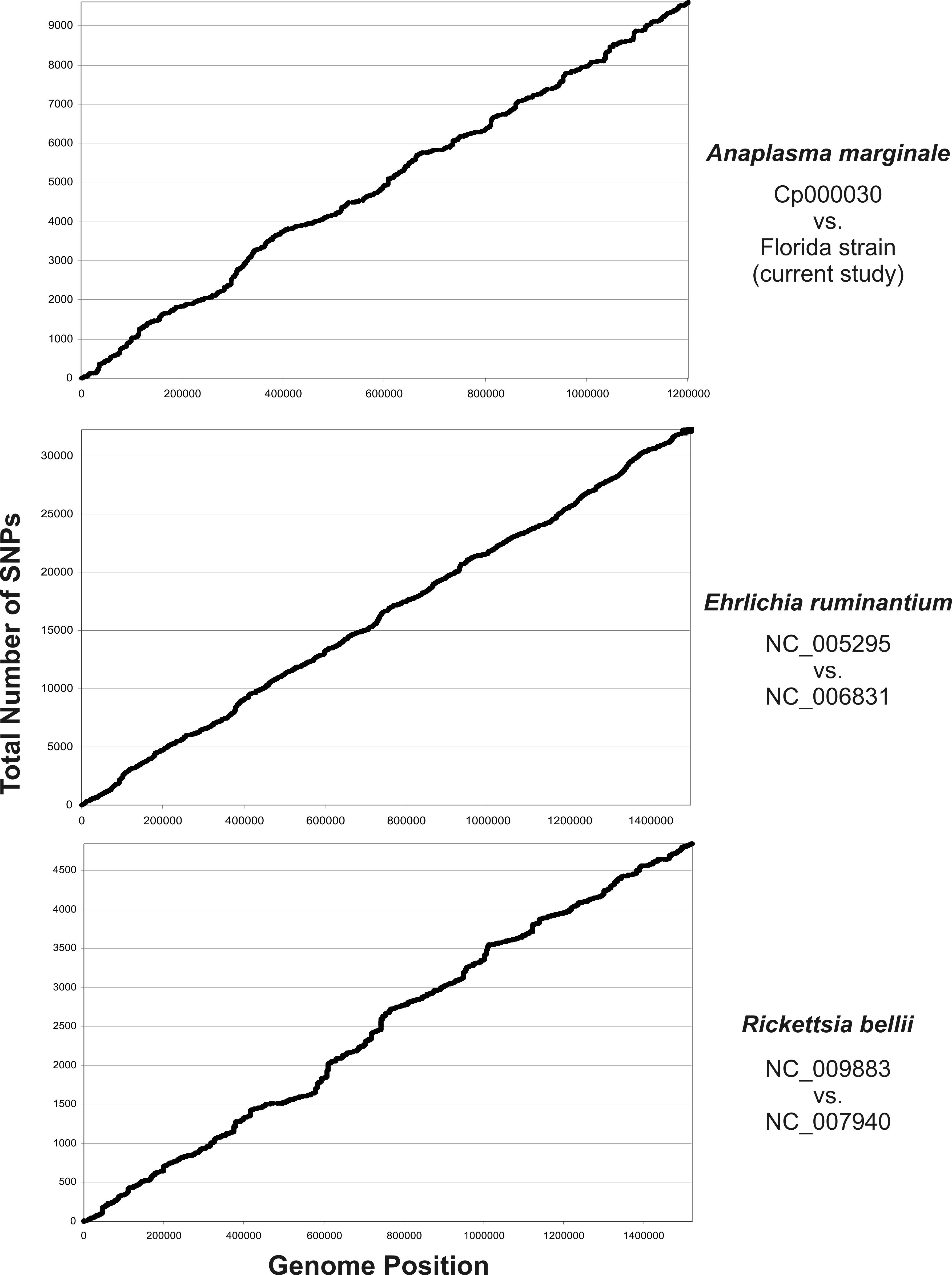

Supplement: Additional file 3 — SNP distribution in three species. Distribution of SNPs between the compared strains of A. marginale, E. ruminantium, and R. bellii. [file 1471-2164-10-16-S3.jpeg]
